# Extraction and Characterization of Natural Cellulosic Fiber from *Pandanus amaryllifolius* Leaves

**DOI:** 10.3390/polym13234171

**Published:** 2021-11-29

**Authors:** Z. N. Diyana, R. Jumaidin, M. Z. Selamat, R. H. Alamjuri, Fahmi Asyadi Md Yusof

**Affiliations:** 1 Fakulti Kejuruteraan Mekanikal, Universiti Teknikal Malaysia Melaka, Hang Tuah Jaya, Durian Tunggal, Melaka 76100, Malacca, Malaysia; nurdiyanazakuan@gmail.com (Z.N.D.); zulkeflis@utem.edu.my (M.Z.S.); 2 Fakulti Teknologi Kejuruteraan Mekanikal dan Pembuatan, Universiti Teknikal Malaysia Melaka, Hang Tuah Jaya, Durian Tunggal, Melaka 76100, Malacca, Malaysia; 3 Faculty of Tropical Forestry, Universiti Malaysia Sabah, Jalan UMS, Kota Kinabalu 88400, Sabah, Malaysia; 4 Malaysian Institute of Chemical & Bioengineering Technology (UniKL MICET), Universiti Kuala Lumpur, Taboh Naning, Alor Gajah, Melaka 78000, Malacca, Malaysia; fahmiasyadi@unikl.edu.my

**Keywords:** *Pandanus amaryllifolius* fibre, natural fibres, composite

## Abstract

*Pandanus amaryllifolius* is a member of Pandanaceae family and is abundant in south-east Asian countries including Malaysia, Thailand, Indonesia and India. In this study, *Pandanus amaryllifolius* fibres were extracted via a water retting extraction process and were investigated as potential fibre reinforcement in polymer composite. Several tests were carried out to investigate the characterization of *Pandanus amaryllifolius* fibre such as chemical composition analysis which revealed *Pandanus amaryllifolius* fibre’s cellulose, hemicellulose and lignin content of 48.79%, 19.95% and 18.64% respectively. Material functional groups were analysed by using Fourier transform infrared (FTIR) spectroscopy and X-ray diffraction analysis confirming the presence of cellulose and amorphous substances in the fibre. The morphology of extracted *Pandanus amaryllifolius* fibre was studied using a scanning electron microscope (SEM). Further mechanical behaviour of fibre was investigated using a single fibre test with 5 kN cell load and tensile strength was found to be 45.61 ± 16.09 MPa for an average fibre diameter of 368.57 ± 50.47 μm. Meanwhile, moisture content analysis indicated a 6.00% moisture absorption rate of *Pandanus amaryllifolius* fibre. The thermogravimetric analysis justified the thermal stability of *Pandanus amaryllifolius* fibre up to 210 °C, which is within polymerization process temperature conditions. Overall, the finding shows that *Pandanus amaryllifolius* fibre may be used as alternative reinforcement particularly for a bio-based polymer matrix.

## 1. Introduction

Changes from the dominant usage of synthetic fibre to natural fibre indicate the rise of awareness among people around the world regarding the negative environmental impact that synthetic fibre brings which may damage human health. Synthetic polymers production utilized a large amount of energy which produces environmental pollutants during the production and recycling of synthetic composites [1]. The implementation of natural fibre-reinforced polymer (NFRP) composites has become an emerging trend driven by stringent environmental legislation that focuses on the development of more eco-friendly materials as an alternative to substitute synthetic composites. The incorporation of natural fibres in composite has presented many improvements in terms of favourable tensile properties, reduced health hazards, acceptable insulating properties, low density and decreased energy consumption [2]. Furthermore, cellulosic material has been widely utilized in many applications such as cellulose nanofibers in food packaging, drug delivery and biomedicine applications; chitosan widely used in biosensors and tissue engineering, membrane separation and treatment of water applications; and hybrid of bacterial cellulose and chitin nanofibers have produced nanocomposite film that provides an excellent barrier to act as the antioxidant and antibacterial film [3]. Besides that, many studies have been conducted with regards to the incorporation of natural fibre in composite applications which have proven that natural fibre has remarkable advantages and is a promising method to replace synthetic polymer [4,5,6,7,8,9].

Natural fibres can be simply defined as fibres that are not synthetic or man-made. They can be sourced from plants, animals or minerals where would be the common classification of natural fibres. In general, most common natural fibres come from plants and are composed of cellulose, thus making the fibre hydrophilic due to the presence of poly(1,4-β-d-anhydroglucopyranose) units which contain hydroxyl groups that enable them to form strong hydrogen bonds [10,11]. They are also called lignocellulosic fibres since their cellulose fibrils are embedded in the lignin matrix [12]. However, natural fibres properties and structural parameters differ among plants depending on the species, growing environment, topographical location and preparation sample fibre method. The plant cell wall is composed of cellulose, hemicellulose and lignin where cellulose is the fundamental composition of the fibre. [12]. Cellulose is an organic polysaccharide that is composed of thousands of d-glucose units resulting from condensation via β(1→4)-glycosidic bonds which permit cellulose to have physical and chemical properties that demonstrate high tensile strength, are environmentally friendly, non-toxic and totally renewable [13]. Hemicellulose is known as a branching polysaccharide polymer made up of glucose and other types of sugar groups including xylose, galactose, arabinos and mannose [11]. Lignin is composed largely of phenylpropane and is the second most abundant component after cellulose, responsible for cementing cellulose microfibrils as well as protecting cellulose and hemicellulose contributes rigidity and also carries out water transport [4]. Both lignin and hemicellulose are amorphous polymers and cellulose is a semicrystalline polymer.

*Pandanus amaryllifolius* is a tropical plant and a member of the Pandanaceae family. It is reported that there are more than 600 known species from the Pandanaceae family [13]. *Pandanus amaryllifolius* is one of the species that is known as an endemic plant to Malaysia and is famously called as pandan wangi. It is famous with regards to its unique fragrant leaves which are widely used for flavouring in the cuisines of the Southeast Asia region. Nevertheless, its species member called *Pandanus tectorius* has flowers that are scented but not the leaves [14]. The distinct flavour aroma from pandan wangi species is due to the presence of a high amount of 2-acetyl-1-pyrroline (2AP) [15]. Despite its unique aroma and colouring, pandan wangi also has been used traditionally as medicine for decades and is proven to enhance the immune system and anti-tumour agents [16]. Besides that, it is also reported that *Pandanus amaryllifolius* extract is able to reduce blood glucose levels as well as shows improvement in insulin resistance [17]. Table 1 shows *Pandanus* species and applications based on reported journals.

Since *Pandanus amaryllifolius* plant has various applications and benefits, final products from extraction process or filtrate are taken out for further processes to leave *Pandanus amaryllifolius* fibre residue as a by-product. The remaining *Pandanus amaryllifolius* residues are usually unused and then discarded as waste. It is reported that about 1 kilogram of dried powder of pandan wangi can yield pandan extract of about 250 g [16]. Thus, if a huge scale of *Pandanus amaryllifolius* extract is produced this will contribute to the larger amount of pandan wangi waste that would be discarded. Moreover, since *Pandanus amaryllifolius* fibre is a source of lignocellulose which is abundant, can be obtained at low cost and is renewable, this would turn it into a suitable candidate for reinforcement agent in composite material application. Hence, it would be waste if the by-product is not fully utilized yet it has good potential to be recycled and able to produce to be another product.

A study reported by Adhamatika et al. [24], characterized physiochemical *Pandanus amaryllifolius* leaves in an attempt to convert the leaves to powder form for a variety of applications. Different parts of pandan leaves were taken; young and old leaves, and undergone three different drying methods; cabinet, vacuum and freeze-drying, prior turned into pandan powder. Although the investigation of physiochemical was conducted, the study has only focused on the non-cellulosic content in the pandan powder including ash content (%), chlorophyll content (mg/g), phenolic content (mg/g) and antioxidant (ppm) and no cellulosic information such as cellulose and hemicellulose content were demonstrated and no mechanical test conducted as the leaves were all converted into powder. Thus, the *Pandanus amaryllifolius* leaves characterization has not been well studied as source of fibre composite. Another study reported by Ooi et al. [25], focused on the potential development of new antiviral protein from purification and characterization of *Pandanus amaryllifolius* leaves. It was observed that the composition of lectin, designated Pandanin, possessed antiviral activities against human viruses namely herpes simplex virus type-1 (HSV-1). The extraction of lectin in the *Pandanus amaryllifolius* leaves was undertaken through a saline extract that underwent a few processes such as monium sulfate precipitation, affinity chromatography and gel filtration. It is interesting to acknowledge on the potential of Pandanin in *Pandanus amaryllifolius* could be a good candidate for a new class of anti-HIV or other antiviral agents. Therefore, it is clear that the reports of both Adhamatika et al. [24] and Ooi et al. [25] do not focus on the fibre characterization as the potential of fibre reinforcement.

Although, there are several studies reported on the potential of *Pandanus amaryllifolius* fibre as composite fillers application such as *Pandanus amaryllifolius* fibre in low-density polyethylene (LDPE) composite [23] and in epoxy resin composite [26]. However, to the best of our knowledge, there has been no research carried out on the characterization of *Pandanus amaryllifolius* fibre that comprehensively focused on its physicochemical and mechanical properties as a potential reinforcement agent. Hence, the study reported in this article focuses on the extraction and characterization of the physical, chemical and mechanical properties of *Pandanus amaryllifolius* fibre for possible utilization as fillers in polymer reinforcement matrix.

## 2. Materials and Methods

### 2.1. Materials

**Figure 1:**
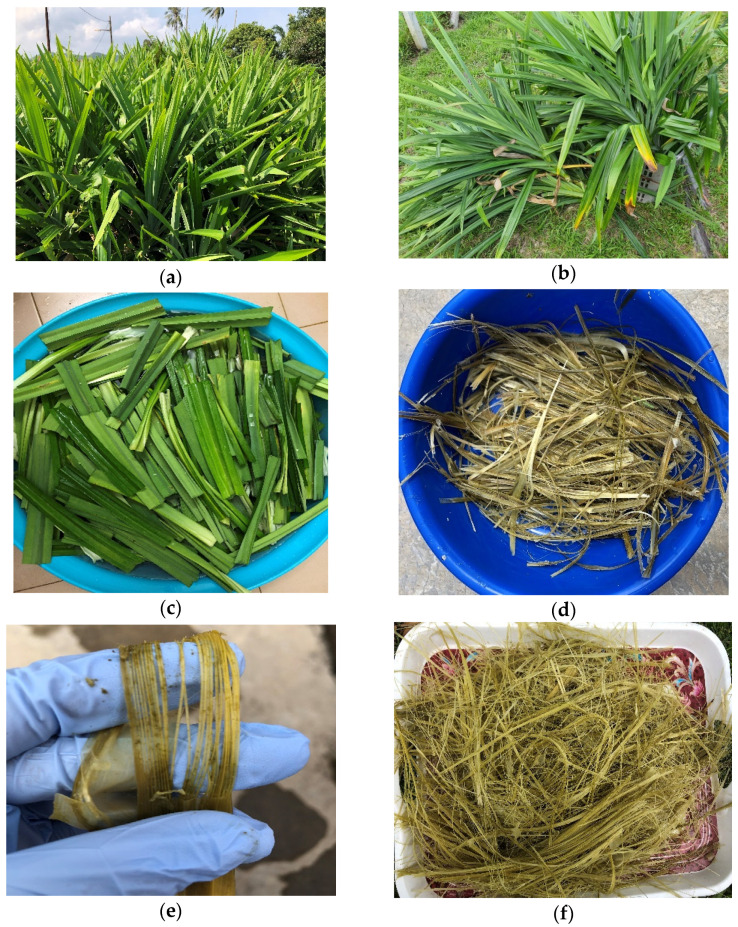

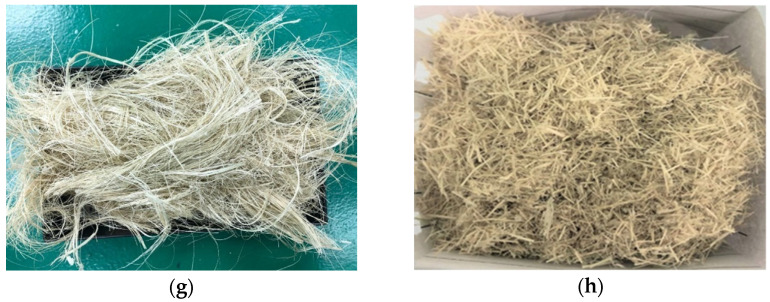
Pictorial view of fibre extraction (**a**) *Pandanus amaryllifolius* plant; (**b**) cut bulk of leaves; (**c**) leaves cleaned and immersed in the water; (**d**) after 2 weeks; (**e**) skin degraded and removal of fibres; (**f**) extracted fibres collected and dried under sunlight; (**g**) ungrounded fibres after being further dried in oven; (**h**) ground (short fibres) PAF.

The *Pandanus amaryllifolius* (pandan wangi) leaves collected from rural area of Bahau (Negeri Sembilan, Malaysia) were used in this study. *Pandanus amaryllifolius* fibre (PAF) were extracted from fresh leaves. Samples were characterized in short length fibre form as shown in Figure 1h except for mechanical testing.

### 2.2. Pandanus amaryllifolius Fibre Extraction

The extraction of PAF involved in this study used the most common fibre extraction technique known as water retting. This technique was carried out by introducing moisture and chemical enzymatic reaction to extract the fibre in the pandan wangi leaves. Initially, bundles of pandan wangi leaves were cleaned and chopped into 12 cm × 3 cm pieces. Then the leaves were then placed in stagnant water to undergo water retting process for 8 weeks. Throughout the process, it was observed that the freshly collected pandan wangi leaves were green in colour at the beginning and changed to yellow-brownish colour after 8 weeks of water retting. The retted leaves were washed in running water and the pandan wangi fibres were removed by manual peeling. The extracted fibres were then cleaned and allowed to dry under direct sunlight for 1 day. After that, the extracted pandan wangi fibres were further dried in the oven because drying under the sunlight is not enough to remove all the moisture in extracted fibres. The fibres were then placed in the oven for 6 h at 80 °C. Some of the fibres were taken and ground using a disc mill to make the short fibre form. Both ground (short fibres) and unground fibres were kept in the zip-locked bag until further use. The images of different steps during the retting process are provided in Figure 1 below.

### 2.3. Characterization

#### 2.3.1. Determination of Chemical Composition

The *Pandanus amaryllifolius* fibres (PAF) chemical composition was evaluated via neutral detergent fibre (NDF), acid detergent fibre (ADF) and acid detergent lignin (ADL), as well as ash content analysis. Using Equations (1) and (2), the cellulose and hemicellulose percentages can be determined respectively:

Cellulose = ADF − ADL(1)

Hemicellulose = NDF − ADF(2)

#### 2.3.2. Fibre Length and Diameter

PAF fibre length was measured from the extracted fibre obtained after water retting and drying. Meanwhile, fibre diameter was evaluated under an optical microscope, Zeiss (Axiovert 200; Carl Zeiss Light Microscopy, Göttigen, Germany) that measured 15 individual fibre samples. The average diameter was randomly measured at three different positions of each image and the average value was determined.

#### 2.3.3. Moisture Content

Five samples were prepared for the moisture content investigation. The weight of the samples before (*W_i_*) was recorded prior to being heated in an oven for 24 h at 105 °C. The final weight after heating (*W_f_*) was recorded after constant weight was obtained to ensure no moisture remained in the sample. The moisture content of the samples was determined using the following Equation (3):

(3) $$\mathrm{Moisture}\;\mathrm{content}\;(\%)=\frac{W_{i}-W_{f}}{W_{i}}\;\times \;100$$

#### 2.3.4. Scanning Electron Microscope (SEM)

Sample’s morphology was observed by using a scanning electron microscope (SEM) machine, model Zeiss Evo 18 Research, (Jena, Germany) with an acceleration voltage of 10 kV.

#### 2.3.5. Mechanical Testing

**Figure 2:**
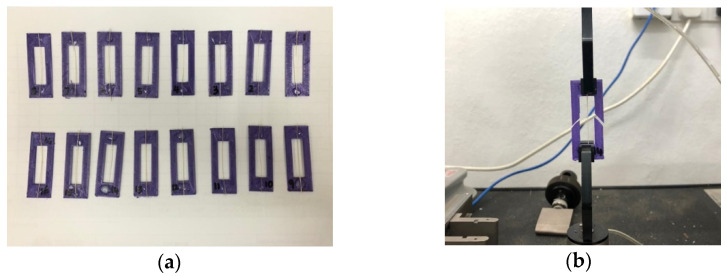
Tensile testing; (**a**) fastening to structure; (**b**) testing assembly.

The ASTM D3379 (1998) guideline was adopted in the evaluation of the tensile properties of PAF by using an Instron universal testing machine (5556, Norwood, MA, USA) with 5 kN load cell capacity. Experimentation was performed with 30 mm gauge length and 1 mm/min crosshead speed. To ensure an adequate fibre fastening to the tensile machine, fibre was glued earlier to a rectangular structure of 20 mm in width and 50 cm in length as shown in Figure 2a and testing assembly is shown as Figure 2b.

#### 2.3.6. Fourier-Transform Infrared (FTIR) Spectrometry

Fourier transform infrared (FT-IR) spectroscopy was used to investigate the intermolecular interaction and presence of functional group of PAF. Spectra of the material were obtained using JASCO FTIR-6100 Spectrometer (Japan). FT-IR spectra of the sample was collected in the range of 4000 to 500 cm^−1^ at resolution of 2 cm^−1^.

#### 2.3.7. Thermogravimetric Analysis (TGA)

Thermogravimetric analysis (TGA) was carried out using a Mettler-Toledo AG analyzer (Greifensee, Switzerland). The test was performed in a temperature range between 30 and 600 °C in inert (nitrogen) atmosphere heating rate of 20 °C/min with a flowrate 20 mL/min. A sample of 5–10 mg of the composite was heated in an alumina crucible pan. Before the thermal study, the samples were pre-conditioned for at least 48 h at 53%.

#### 2.3.8. X-ray Diffraction (XRD) Analysis

The X-ray diffraction was conducted using a Rigaku D/max 2500 X-ray powder diffractometer (Rigaku, Tokyo, Japan) with Cu radiation run at 40 kV and 30 mA with 0.15406 nm light wavelength. The scanning rate of 2° min^−1^ in the range of diffraction angle 10° to 40° at room temperature was used to scan the samples. The crystallinity index (*CI*) was computed using the subsequent Segal expression, Equation (4):

(4) $$CI=\frac{I_{002}-I_{am}}{I_{002}}\times 100\%$$

where *I*_002_ and *I_am_* represent peak intensities of the crystalline and amorphous fractions, respectively. The crystallite size (*CS*) was determined using Scherrer’s formula as shown in Equation (5),

(5) $$\;CS=\frac{k\lambda \;}{\beta \cos\theta }\times 100\%$$

where *k* = 0.89 (Scherrer’s constant), *λ* = 0.1541 nm is the radiation wavelength, β is the peak’s full-width at half-maximum in radians, and θ is the corresponding Bragg angle.

## 3. Results and Discussion

### 3.1. Determination of Chemical Properties

Table 2 presents the chemical composition of *Pandanus amaryllifolius* fibre in comparison with other natural fibres. Mechanical properties and biodegradability of fibre depend on its chemical composition [27]. From Table 2, *Pandanus amaryllifolius* fibre shows value of cellulose content of 48.79% comparable to sugarcane fibre and *Arenga pinnata* fibre, 48% and 43.88% respectively, and shows lower cellulose content than other natural fibres such as *Calotropis gigantea*, *Ficus religiosa*, *Coccinia grandis*, bamboo, jute, sisal, kenaf and acacia tortilis with cellulose content of 64.47%, 55.58%, 63.22%, 73.83%, 66%, 65%, 72% and 61.89% respectively. Meanwhile, cassava bagasse, *Pandanus tectorius* and *Tridax procumbens* show lower cellulose value of 27%, 37.3% and 32% respectively. High cellulose content in fibre contributes to the enhancement of mechanical properties [11]. However, from the data tabulated in Table 2, *Pandanus amaryllifolius* fibre exhibits considerably low cellulose content that might be reflected in the low tensile value obtained in mechanical testing, besides the influence of fibre diameter. This also might be attributed to the presence of a high content of amorphous substances in *Pandanus amaryllifolius* fibre such as hemicellulose, lignin, ash and waxes [28].

The biological role of hemicellulose is to strengthen plant cell walls by interaction with cellulose. *Pandanus amaryllifolius* fibre hemicellulose content of 19.95% is comparable to kenaf fibre, 20.3%, and jute fibre, 17%. It has relatively high hemicellulose content as compared to all other natural fibres except for kenaf, cassava bagasse and *Pandanus tectorius* with hemicellulose content of 20.3%, 30% and 34.4%, respectively. Besides that, the presence of hydrophobic substances such as lignin makes it less susceptible to moisture as well as providing better fibre stability and rigidity; however, it lowers the fibre tensile strength. The lignin content in *Pandanus amaryllifolius* fibre was 18.64% making it comparable to *Acacia tortilis*, 21.26% and *Pandanus tectorius*, 22.6%. It is also seen to have higher lignin content than bamboo, sugarcane, jute, sisal, kenaf and cassava bagasse at 10.5%, 12.1%, 12.5%, 9.9%, 9% and 2.7% respectively. Ash content in *Pandanus amaryllifolius* fibre is recorded at 1.08%, considered as among of the lowest in comparison to other natural fibres tabulated in Table 2 which determines it has less susceptibility to moisture when applied in a humid environment. The value is in good agreement with the result obtained from moisture content testing.

All chemical compositions exhibited in every plant natural fibre have their main functionality and role in maintaining the rigidity of the plant structure and they complement every constituent. However, it is worth noting that in filler composite application, the amount of cellulose in fibre becomes the main criteria that might influence the mechanical properties of the resulting composite.

### 3.2. Determination of Physical Properties

A total of 15 samples of *Pandanus amaryllifolius* fibre was picked randomly to determine the average fibre length. The extracted fibre were chosen in dried condition after undergoing the drying process as presented in Figure 1g. The average ultimate fibre length was found in this study to be 45.07 ± 4.81 mm long (coefficient of variation, CV = 0.11). The coefficient of variation value shows the variability in the individual reading is small meaning the sample is fairly homogenous. In general, fibre physical properties are varied depending on the climate, environmental condition, age and origin of fibre [11]. Besides, the *Pandanus amaryllifolius* fibres were collected from rural areas living alongside other wild plants as displayed in Figure 1a. Physically, it can be observed that the collected fibre is bigger and longer in size compared to *Pandanus amaryllifolius* fibres that are cultivated in residential areas.

**Figure 3:**
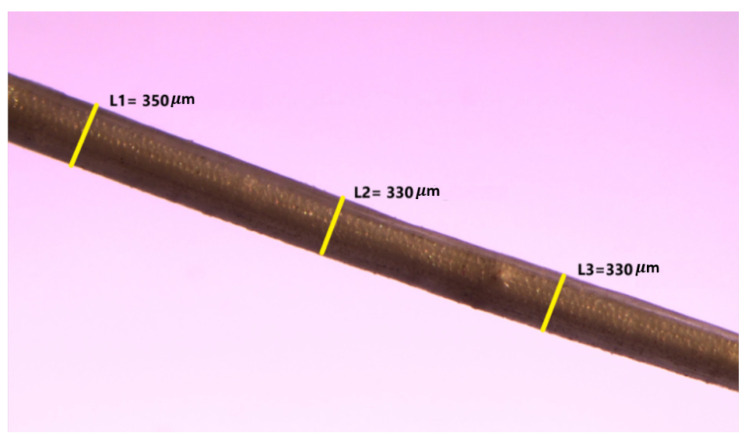
Optical microscopy image of the *Pandanus amaryllifolius* fibre.

Table 3 presents the diameter and moisture content of *Pandanus amaryllifolius* fibre compared to other natural fibres. Fibre diameter plays a vital role in determining fracture surface area thus reflecting the tensile value of the material in determining mechanical properties of the fiber. Thus, the diameter measurement of *Pandanus amaryllifolius* fibre is conducted under an optical microscope as displayed in Figure 3. Fibre average diameter was found to be 368.57 ± 50.47 μm which is comparable to the diameter of *Cissusquadrangularis* root (350 μm) and *Coccinia grandis* (543 ± 75 μm).

Furthermore, moisture content of *Pandanus amaryllifolius* fibre was the lowest among natural fibres as shown in Table 3. However, the moisture content of *Pandanus amaryllifolius* fibre is seen to be the lowest among others and comparable to *Acacia tortilis* and bamboo at 6.47% and 7.00%, respectively. Meanwhile, the moisture content of *Tridax procumbens* fibre showed the highest which at 11.20%. Moisture content plays an important role in selecting the best properties of polymer products such as plastic packaging applications. Thus, the lower moisture content is preferable as it reflects the hydrophobicity of the fibre material.

### 3.3. Mechanical Testing

**Figure 4:**
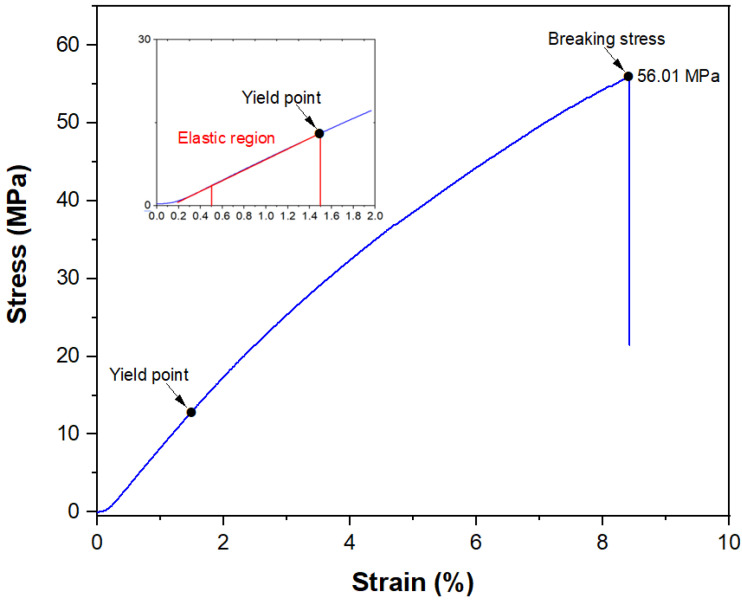
Typical stress-strain curve for *Pandanus amaryllifolius* fibre with magnification of elastic region.

A total of 15 samples of fibre were chosen randomly to be tested under tensile test at a gauge length of 30 mm before obtaining the mechanical properties of the *Pandanus amaryllifolius* fibre. Figure 4 illustrates a typical stress-strain curve behaves quasi-linearly presented an initial elastic region with a strong slope, (*ε* ≤ 1.5%), continued with non-linear elastic region between 2% to 5% strain and reached final softening phase up to 8.3% maximum strain value. Besides, the elastic modulus was calculated from the elastic region values raging between 0.5% and 1.5% as shown in Figure 4. In general, the fibre demonstrated brittle properties due to the sudden drop in stress value in the stress-strain curve which is in good agreement with other reported studies on natural fibres such as *Tridax procumbens* [31], *Juncus effsus* L. [39] and *Agave americana* L. [42]. Typical yield point and breaking stress of *Pandanus amaryllifolius* fibre is labelled in Figure 4 and the elastic region is found to be smaller than the plastic region which is similar to the stress-strain curve obtained for *Juncus effsus* L. [39], *Agave americana* L. [42] and *Vernonia eleagnifolia* [43]. Unlike synthetic fibre, where the size and properties of the produced fibre can be controlled during the manufacturing process before being applied, natural fibre exhibits variation in diameter for every single fibre strand obtained. Therefore, the average value of mechanical tensile properties is displayed in Table 4 with standard deviation to show the variability in individual reading being small, and the material is fairly homogenous compared to tensile results extracted from different research studies.

Sanyang et al. [12] found that the high cellulose content of fibre has a strong influence on their mechanical properties and different parts of the plant contribute to the different amounts of cellulose content from the fibre extracted. The study mentioned that sugar palm fibre extracted from the frond part contained the highest cellulose content (66.49%) as compared to bunch (61.76%) and trunk (40.56%) which leads to the highest fibre strength being found at the frond part (421.4 MPa) rather than the bunch (365.1 MPa) and trunk (198.3 MPa). Therefore, this could relate from data tabulated in Table 2 and Table 4, since *Pandanus amaryllifolius* is a leaf plant and tensile strength obtained is considerably lower than others which might be due to the cellulose content of fibre itself. A similar finding has also been reported on the enhancement of mechanical properties as the cellulose content increased after chemical treatment on fibre was conducted [27]. In general, from Table 4, the average tensile strength, elastic modulus, and elongation value of *Pandanus amaryllifolius* was found to be 45.61 ± 16.09 MPa, 0.41 ± 0.18 GPa, and 8.17 ± 3.84%, respectively. The tensile value found in the present work is higher than *Cymbopogan citratus*, 43.81 ± 15.27 MPa [29] and *Tridax procumbens*, 25.75 MPa [31] which might be attributed to higher cellulose content found in *Pandanus amaryllifolius* fibre (48.79%) as compared to *Cymbopogan citratus* (37.56%) and *Tridax procumbens* (32.00%). Meanwhile, Dawit et al. [27] reported better tensile strength of *Acacia tortilis*, 71.60 MPa than *Pandanus amaryllifolius* which also could relate to higher cellulose content found at 61.89%.

In the meantime, elastic modulus calculated from this study demonstrated an almost similar value to 0.94 ± 0.09 GPa obtained from fibre named *Tridax procumbens* as reported by Vijay et al. [31] and slightly lower than 1.04 ± 0.33 GPa reported for *Cymbopogan citratus* [29]. Moreover, *Pandanus amaryllifolius* fibre elongation at break is found to be an almost similar value of 8.74 ± 1.8% to *Ficus Religiosa* fibre studied by Arul et al. [32] while remaining higher than other fibres such as *Cymbopogan citratus* (0.84 ± 0.28%), *Tridax procumbens* (2.77 ± 0.27%), *Acacia tortilis* (1.30%), *Juncus effuses* L. (2.75 ± 0.6%), *Vernonia eleagnifolia* (6.9%) and date palm (3.6%). In general, although the overall mechanical properties of *Pandanus amaryllifolius* fibre as tabulated in Table 4 is averagely lower than other lignocellulosic fibres, it still can be considered as alternative reinforcement especially for a bio-based polymer matrix which is typically use for low strength application such as biodegradable packaging material.

### 3.4. Scanning Electron Microscope (SEM)

**Figure 5:**
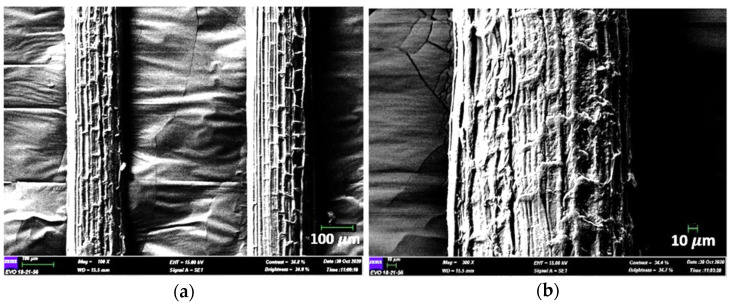
Longitudinal view of *Pandanus amaryllifolius* (pandan wangi) fibre by scanning electron microscope (SEM); (**a**) 100× magnification; (**b**) 300× magnification.

The SEM of PAF at magnification of 100× and 300× are shown in Figure 5a,b, respectively. In general, the surface micrograph of PAF is not smooth and is uneven with the deposition of other substances. From the presented longitudinal image at 100× magnificent, it is observed that the fibre structure exhibits vertical alignments in the same direction of the fibre axis. This is might be attributed to the structure of PAF that is composed of microfibrils, compactly aligned and bonded together by lignin, pectin and other non-cellulosic materials [4]. When the image is zoomed in at 300× magnification, Figure 5b, the structure of microfibrils can be seen and further prove the presence of lignin that is responsible for cementing cellulose microfibrils and protecting the structure. Similar results were reported by other authors [38,45]. Other than that, we also found shapes of regularly distributed semi-rectangular trays exist together along with PAF surface lines. The phenomenon might be exhibited in the deposition of amorphous substances including hemicellulose and lignin and might also be impurities that present during the binding process [40]. Nevertheless, the serration and uneven fibre surface somehow can help improve mechanical performance. It helps in enhancing the fibre-matrix interface in polymeric composite material, locking the fibre mechanically in the polymeric resin [45].

### 3.5. Fourier-Transform Infrared (FTIR) Spectrometry

**Figure 6:**
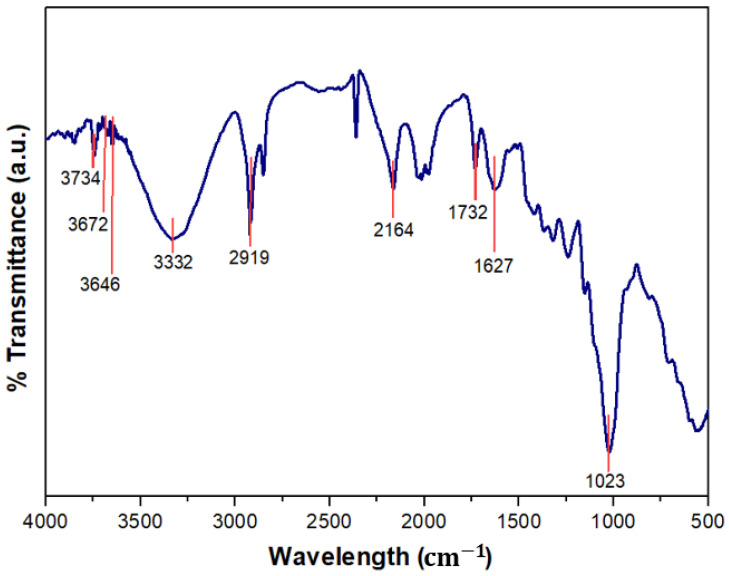
Fourier transform infrared (FTIR) spectra of *Pandanus amaryllifolius* fibre.

The FTIR curve of *Pandanus amaryllifolius* fibre is displayed in Figure 6. The broadband and peak occurred at 3332 cm^−1^ reflecting water adsorption and the presence of the hydroxyl (O–H) group in the fibre which also indicates the existence of cellulose, lignin and water [45]. The presence of water in the fibre was revealed by moisture content tests. Meanwhile, the absorbance peak in the 2919 cm^−1^ and 1732 cm^−1^ represents the stretching of alkanes (C–H) and carboxyl group (C=O) of both cellulose and hemicellulose [38]. The presence of (C=C) stretch from aromatic lignin is observed at wave peaks of 1672 cm^−1^. Besides that, the peak range of 1149–1414 cm^−1^ in the *Pandanus amaryllifolius* fibre spectrum is associated to the (C=O) stretching vibration of ester linkage of the carboxylic group of ferulic and p-coumaric acids of hemicellulose and lignin [46]. Constituents of polysaccharide in cellulose confirm the stretching vibration of (C–O) and (O–H) that occurred at the radiation of 1023 cm^−1^. FTIR analysis verified the existence of cellulose and hemicellulose in the fibre’s structural composition.

### 3.6. X-ray Diffraction Measurement

**Figure 7:**
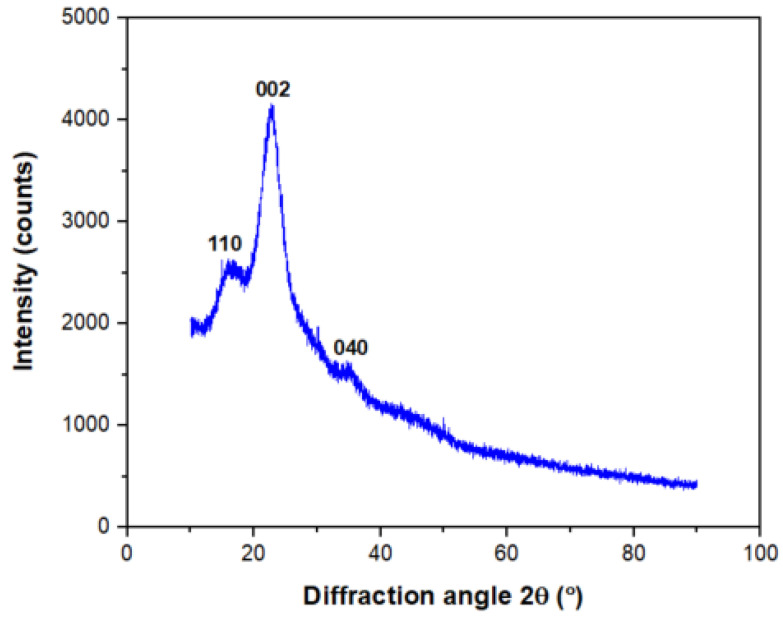
X-ray diffraction pattern of *Pandanus amaryllifolius* fibre.

The diffraction pattern for *Pandanus amaryllifolius* fibre is shown in Figure 7 and the presence of three peaks can be observed. The highest intensity peak is found at 2θ = 23.16°, average density peak at 2θ = 16.78° and the lowest intensity peak occurred at 2θ = 34.69°, which are denoted as the (002), (110) and (040) crystallographic planes, respectively [40]. The highest peak intensity (002) corresponds to the presence of semi-crystalline structure cellulose type I, associated to the parallel chain orientation with intra-sheet hydrogen bonds [47]. Meanwhile, the diffracted peak at intensity (110) proves the amorphous fraction in *Pandanus amaryllifolius* fibre [41]. This result is in corroboration with chemical and FTIR analysis results that confirm the presence of cellulose and amorphous substances.

High crystallinity values stipulate excellent mechanical properties of the fibre which can be identified from the sharpness of the diffraction peak where the sharper diffraction peak indicates a high crystallinity degree of the structure that might be influenced by the cellulose rigidity [48]. Segal’s peak equation Equation (4) is employed to find the crystallinity index (CI) from the peaks found. The CI for *Pandanus amaryllifolius* fibre are calculated found to be 37.09% and 4.95 nm. This CI value is comparable to *Calotropis gigantea* (36.00%), higher than *Tridax procumbens* (34.46%) and *Juncus effuses* L. (33.40%), but lower than *Ficus religiosa* (42.92), *Pandanus tectorius* (55.10%), Cissusquadrangularis root (56.60%), pigeon pea plant (65.89%) and date palm rachis (69.77%).

Crystalline size (CS) for *Pandanus amaryllifolius* fibre is calculated using Segal’s Equation (5) and found to be 4.95 nm. This value is within crystallite range size reported for other natural fibres such as *Ficus religiosa* (5.18 nm), *Tridax procumbens* (25.04 nm) and *Juncus effuses* L. (3.60 nm). The crystalline property of *Pandanus amaryllifolius* fibre is compared with the tested natural fibres shown in Table 5.

### 3.7. Thermogravimetric Analysis

**Figure 8:**
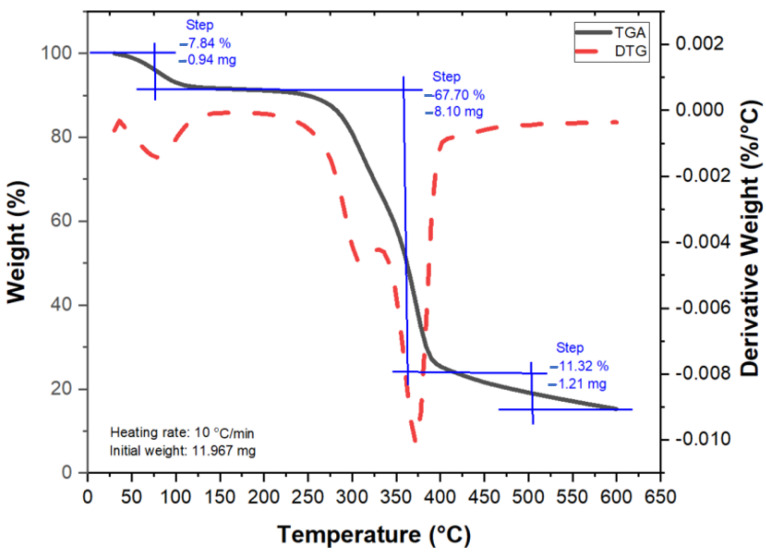
Thermogravimetric analysis (TGA) and DTG curves for *Pandanus amaryllifolius* fibre.

Figure 8 shows thermogravimetric analysis for *Pandanus amaryllifolius* fibre, both TGA and DTG curves were plotted as a function of temperature. This thermal analysis is used to measure weight degradation as the material is heated. In general, three degradation phases can be observed in Figure 8 which comprises small initial weight loss at the temperature range of 30–120 °C, major weight loss as the temperature rises from 220–420 °C, and final stage degradation afterwards until the maximum temperature was reached. The first stage with 7.84% weight loss corresponds to the evaporation of adsorbed moisture which is attributed to the hydrophilic nature of lignocellulosic materials [11,40]. After that, thermal stability is noted up to about 210 °C which reflects no appearance of significant peaks in both TGA and DTG curves showing that the work is in good agreement [39]. Then, major weight loss is observed, 67.70%, associated to the decomposition of glycosidic linkage cellulose and hemicellulose substances at the temperature range of 220–420 °C [41]. It is confirmed by the observation of the DTG peak curve at 370 °C which indicates the possible thermal decomposition of cellulose I and completion of α-cellulose decomposition [33]. Lastly, the final stage of degradation is associated to the fragmentation of waxen substances such as lignin and observed at temperatures 420–500 °C with small weight loss of 11.32%. Last but not least, char residue is spotted at 13.14% at the highest temperature of 600 °C. This char residue is mainly composed of carbon or carbonaceous material that cannot be further degraded into smaller volatile fragments and will remain in the combustion chamber until the process ends. It also might be attributed to the presence of inorganic matter that yields chars and may form the basis of quantitative estimation. Boumediri et al. [40] and Maache et al. [39] reported on char residue left at the end of combustion of an amount of 3.64% for *Juncus effuses* L. fibre and 18.29% for date palm fibre, respectively.

## 4. Conclusions

The *Pandanus amaryllifolius* fibres were successfully extracted from plants via a water retting extraction process. The *Pandanus amaryllifolius* fibres were characterized for physical, thermal, chemical and mechanical properties. The chemical composition analysis revealed that *Pandanus amaryllifolius* fibre has cellulose, hemicellulose, and lignin content of 48.79%, 19.95% and 18.64%, respectively. FT-IR and XRD analysis confirmed the presence of cellulose and amorphous substances in the fibre. The tensile strength of the fibre was 45.61 ± 16.09 MPa for an average fibre diameter of 368.57 ± 50.47 μm. Moisture content analysis indicated 6.00% moisture content of *Pandanus amaryllifolius* fibre. The thermogravimetric analysis showed the thermal stability of *Pandanus amaryllifolius* fibre up to 210 °C, which is within polymerization process temperature conditions. Overall, the finding shows that *Pandanus amaryllifolius* fibre may be used as alternative reinforcement particularly for a bio-based polymer matrix. Furthermore, utilization of *Pandanus amaryllifolius* fibre as the reinforcing agent will lead to greater development of more biopolymer products since this natural fibre can be reinforced with a synthetic and natural polymer matrix.

## Figures and Tables

**Table 1:** Pandan species and potential applications.

Type of Pandan	Potential Application	References
*Pandanus ceylanicus*	Polymer composite	[13]
*Pandanus tectorius*	Natural fiber	[18]
*Pandanus utilis*	Low cost paper	[19]
*Pandanus amaryllifolius*	Polymer composite	[20,21]
*Pandanus tectorius*	Pharmaceutical, medicine	[16]
*Pandanus amaryllifolius*	Pharmaceutical, medicine	[17]
*Pandanus odoratissimus*	Traditional medicine	[22]
*Pandanus amaryllifolius*	Packaging application	[23]

**Table 2:** Comparative chemical composition of *Pandanus amaryllifolius* fibre and other natural fibres.

Fibre	Cellulose (%)	Hemicellulose (%)	Lignin (%)	Ash (%)	Ref.
*Pandanus amaryllifolius*	48.79	19.95	18.64	1.08	-
*Cymbopogan citratus*	37.56	29.29	11.14	4.28	[29]
*Calotropis gigantea*	64.47	9.64	13.56	3.13	[30]
*Tridax procumbens*	32.00	6.80	3.00	0.71	[31]
*Ficus religiosa*	55.58	13.86	10.13	4.86	[32]
*Coccinia grandis*	63.22	-	24.42	-	[33]
Bamboo	73.83	12.49	10.5	-	[34]
Sugarcane	48.00	14.60	12.10	12.10	[35]
Jute	66.00	17.00	12.50	-	[36]
Sisal	65.00	12.00	9.90	-	[36]
Kenaf	72.00	20.30	9.00	-	[36]
Cassava bagasse	27.00	30.00	2.70	-	[37]
*Acacia tortilis*	61.89	-	21.26	4.33	[27]
*Arenga pinnata* (sugar palm)	43.88	7.24	33.24	1.01	[38]
*Pandanus tectorius*	37.30	34.40	22.60	24.00	[11]

**Table 3:** Comparative physical properties of *Pandanus amaryllifolius* fibre and other natural fibres.

Fibre	Diameter (μm)	Moisture (%)	Ref.
*Pandanus amaryllifolius*	368.57 ± 50.47	6.00 ± 0.13	-
*Cymbopogan citratus*	326.67 ± 45.77	5.20 ± 2.28	[29]
*Calotropis gigantea*	32.70	7.27	[30]
*Tridax procumbens*	233.1 ± 9.9	11.20	[31]
*Juncus effuses* L.	280 ± 56	-	[39]
*Ficus Religiosa*	25.62	9.33	[32]
Date palm rachis	88 ± 12	-	[40]
*Coccinia grandis*	543 ± 75	9.14	[33]
*Cissusquadrangularis* root	350	7.30	[41]
Bamboo	-	7.00	[34]
*Acacia tortilis*	480	6.47	[27]
*Arenga pinnata* (sugar palm)	212.01 ± 2.17	8.36	[38]

(Note: Coefficient of variations of fibre diameter for *Pandanus amaryllifolius* fibre, CVFD = 0.14).

**Table 4:** Comparison tensile properties of *Pandanus amaryllifolius* fibre with other natural fibre.

Fibre	Tensile Strength, *σ* (MPa)	Tensile Modulus, E (GPa)	Elongation at Break, *ε* (%)	Ref.
*Pandanus amaryllifolius*	45.61 ± 16.09	0.41 ± 0.18	8.17 ± 3.84	-
*Cymbopogan citratus*	43.81 ± 15.27	1.04 ± 0.33	0.84 ± 0.28	[29]
*Tridax procumbens*	25.75	0.94 ± 0.09	2.77 ± 0.27	[31]
*Acacia tortilis*	71.63	4.21	1.33	[27]
*Juncus effuses* L.	113 ± 36	4.38 ± 1.37	2.75 ± 0.6	[39]
*Vernonia eleagnifolia*	259.6	37.8	6.9	[43]
*Ficus Religiosa*	433.32 ± 44	5.42 ± 2.6	8.74 ± 1.8	[32]
*Coccinia grandis*	424.24	26.52	16.00	[33]
Date palm	530.5	21.9	3.6	[40]
Bamboo	583.0	25.5	2.1	[44]
Kenaf	393–773	26.5	1.6	[36]
Sisal	511–635	2.2–9.4	1.5	[36]
Kenaf	930.0	53.0	1.5	[36]

**Table 5:** Comparison table of crystalline properties of *Pandanus amaryllifolius* fibre with other natural fibres.

Fiber	C.I (%)	C.S (nm)	References
*Pandanus amaryllifolius*	37.09	4.95	-
*Cymbopogan citratus*	35.20	-	[29]
*Calotropis gigantea*	36.00	-	[30]
*Tridax procumbens*	34.46	25.04	[31]
*Juncus effuses* L.	33.40	3.60	[39]
*Ficus Religiosa*	42.92	5.18	[32]
*Pandanus tectorius*	55.10	-	[11]
*Cissusquadrangularis* root	56.60	7.04	[41]
Pigeon pea plant	65.89	11.60	[49]
Date palm rachis	69.77	5.63	[40]

## Data Availability

The data presented in this study are available on request from the corresponding author.
